# Analysis of DNA topology of EBV minichromosomes in HEK 293 cells

**DOI:** 10.1371/journal.pone.0188172

**Published:** 2017-11-29

**Authors:** Alicia Castán, Vanessa Fernández-Calleja, Pablo Hernández, Dora B. Krimer, Jorge B. Schvartzman, María-José Fernández-Nestosa

**Affiliations:** 1 Department of Cellular and Molecular Biology, Centro de Investigaciones Biológicas (CSIC), Ramiro de Maeztu 9, Madrid, Spain; 2 Scientific and Applied Computing Laboratory, Polytechnic School, National University of Asunción, SL, San Lorenzo, Paraguay; University of North Carolina at Chapel Hill, UNITED STATES

## Abstract

Simian Virus 40 (SV40) and Epstein-Barr Virus (EBV) are frequently used as model systems to study DNA replication. Their genomes are both circular duplex DNAs organized in a single replicon where replication initiates at a precise site upon binding of a specific protein: the large tumor (T) antigen for SV40 and the Epstein-Barr Nuclear Antigen 1 (EBNA-1) for EBV. Despite the abundant information available on the genetics and biochemistry of the replication process in these systems, little is known about the changes in DNA topology that take place as molecules are transfected into eukaryotic cells, assembled into chromatin and bind initiator proteins to start replication. Here we used high-resolution two-dimensional agarose gel electrophoresis to demonstrate that in Human Embryonic Kidney (HEK) 293 cells, minichromosomes of almost the same mass carrying either the SV40 or the EBV replication origin showed similar topological features. The patterns were very similar regardless of the initiator proteins. We also showed that in a hybrid minichromosome, pEco3’Δ, that initiates replication from the SV40 origin, the presence of EBNA-1 and its putative binding to the EBV “family of repeats” induces no significant topological change. These observations challenge the idea that binding of EBNA-1 to *oriP* could induce negative supercoiling and favor a model suggesting that it binds to *oriP* in a two-step process where only the second step causes structural changes in a transient cell cycle specific manner.

## Introduction

Duplication of the Simian Virus 40 (SV40) and the Epstein-Barr Virus (EBV) genomes in eukaryotic cells are probably the best characterized replication processes to date and are frequently used as model systems to study DNA replication [1–5]. SV40 and EBV genomes are circular duplex DNAs organized in a single replicon. The SV40 genome is 5,243 bp long and encodes the single protein required to initiate its replication: the SV40 large tumor (T) antigen [6]. Although originally found in monkeys (SV40 is an abbreviation for Simian Vacuolating Virus 40), this polyomavirus is also found in humans. Derivatives of SV40 DNA initiate bi-directional replication *in vitro* in soluble extracts prepared from human HEK 293 cells supplemented with the SV40 T-antigen provided they contain the SV40 origin of replication [7].

The EBV genome is significantly larger with a length of around 172,000 bp encoding about 85 genes [3]. It is maintained extra-chromosomally in infected human cells and in order to initiate replication requires a single protein named Epstein-Barr Nuclear Antigen 1 (EBNA-1). Surprisingly, a specific contiguous sub-fragment of EBV DNA of about 1,700 bp supports autonomous extra-chromosomal replication when EBNA-1 is provided *in trans*. This sub-genomic EBV DNA is termed *oriP*, for “origin of plasmid replication” [8]. *oriP* contains two relevant components: a “dyad symmetry” (DS) element and an array of tandem repeats, termed the “family of repeats” (FR) located about 1 Kbp upstream the DS. The FR consists of 21 imperfectly conserved, 30 bp direct repeats that include sequence motifs to which EBNA-1 binds with high affinity. These motifs are also present in the 120 bp DS element [3]. Initiation of DNA replication at *oriP* is thought to occur at or near the DS element in a bi-directional manner. Several regions within *oriP* are known to melt easily probably due to its high A+T content [9]. This melting is thought to be stabilized by (-) supercoiling, although binding of Cdc6 and Orc was found to partially suppress the EBNA-1 induced sensitivity to single-stranded endonucleases [10]. In any case, the FR acts as a replication fork barrier (RFB) and termination site. Thus, the replication of EBV minichromosomes proceeds in a predominantly uni-directional manner [11].

Despite the abundant information on their replication modes and requirements [1–5] little is known about how the topology of the DNA changes as the molecules are transfected into eukaryotic cells, assembled into chromatin and bind initiator proteins to start replication. This is particularly intriguing since SV40 and EBV were among the first viral DNAs where DNA supercoiling and catenation were originally discovered [12–15]. Moreover, Keller [16] was the first to use agarose gel electrophoresis combined with intercalating agents to determine the exact number of superhelical turns in SV40 DNA.

Supercoiled recombinant plasmids isolated from bacteria are frequently used to transfect eukaryotic cells [17]. There are significant differences, though, in the organization of the DNA before and after transfection. In prokaryotes DNA is not regularly wrapped around proteins and supercoiling is regulated by the opposing action of DNA gyrase and topoisomerase I. In eukaryotes, on the other hand, most of the DNA supercoils are constrained within nucleosomes [18, 19]. It is well known that shuttle vectors that can efficiently replicate in both bacteria (*Escherichia coli*) and yeast (*Saccharomyces cerevisiae*) are more (-) supercoiled in bacteria [20]. The assembly of transfected bacterial DNA into eukaryotic chromatin involves two independent processes: relaxation of the bacterial native (-) supercoiling by eukaryotic topoisomerases 1 and 2 and its association with histone complexes (nucleosomes) to form chromatin. It was repeatedly shown that this latter process is fully accomplished in cultured cells about 36 hours after transfection and DNA replication is not required [21, 22].

**Fig 1:**
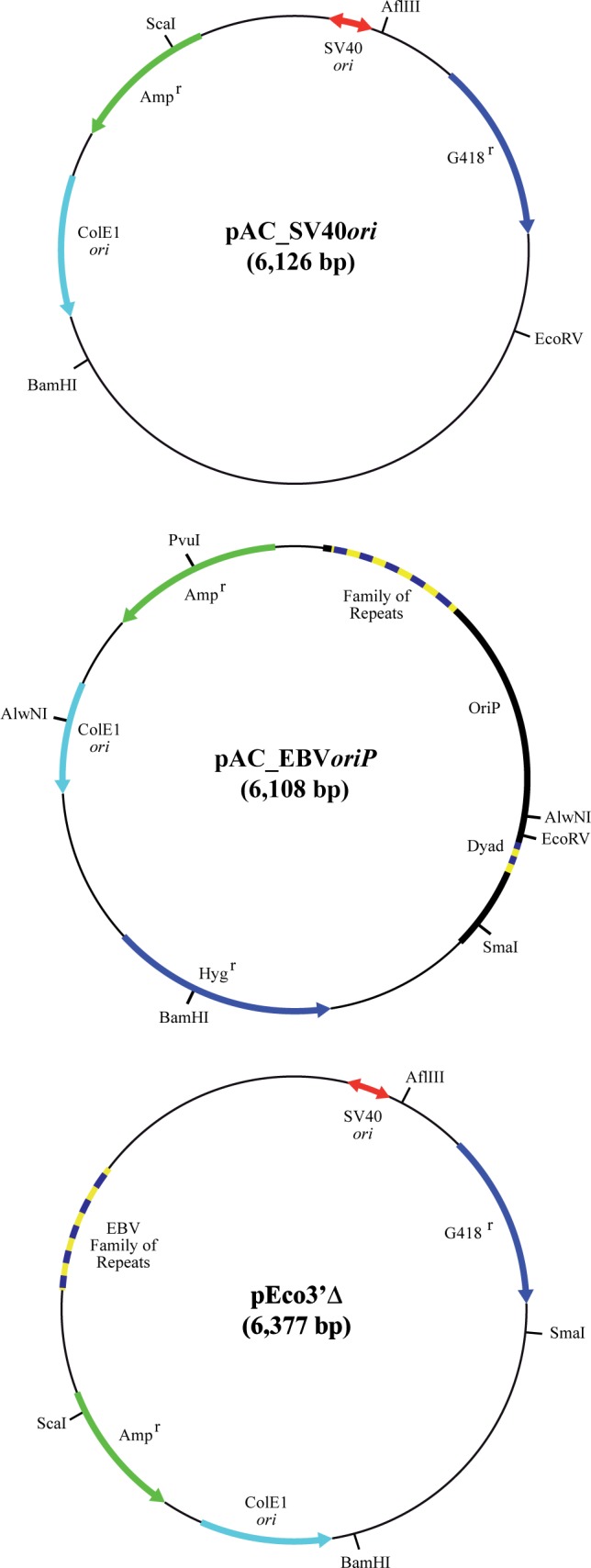
Genetic maps of the minichromosomes used in this study. On top pAC_SV40*ori* (6,126 bp) that contains the SV40 bi-directional origin of replication (outlined in red) showing its most relevant elements. In the middle pAC_EBV*oriP* (6,108 bp) that contains the EBV bidirectional origin of replication (*oriP*), including the “dyad symmetry” element (DS) and the “family of repeats” (FR). At the bottom, pEco3’Δ (6,377 bp) containing the SV40 bi-directional origin of replication (in red) and the EBV’s FR (outlined in blue and yellow).

We wanted to know if the different replication initiation modes of SV40 and EBV minichromosomes in human cells were related to changes in DNA topology. To this end we took advantage of HEK 293 original and variant cells that could constitutively express either the SV40 large T- antigen (HEK 293T) or the Epstein-Barr Nuclear Antigen 1 (HEK 293E). We transfected HEK 293T cells with a derivative of SV40, pAC_SV40*ori* and HEK 293 as well as HEK 293E cells with a derivative of EBV, pAC_EBV*oriP* (Fig 1). About 40 hours after transfection non-replicating extra-chromosomal DNA was isolated and the minichromosomes were analyzed by high-resolution two-dimensional agarose gel electrophoresis (2D gels) where the first dimension occurred without or in the presence of different concentrations of chloroquine. We examined also another recombinant minichromosome, pEco3’Δ, that contains the SV40 replication origin and the FR of EBV’s *oriP* but not the DS element (Fig 1). The topology of this minichromosome was studied in HEK 293T cells and in the same cells co-transfected with pEco3’Δ and another minichromosome, pCXWB-EBNA-1, that expresses EBNA-1. The results obtained clearly demonstrated that the presence of EBNA-1 and its putative binding to the FR has no apparent topological consequence.

## Material and methods

### Recombinant plasmids

Plasmids used in this study were: 1) pAC_SV40*ori*, a derivative of pEco3’Δ, supplied by C. Schildkraut [23], obtained after removal of the *Eco*RI-*Xho*I 1,680 bp fragment corresponding to the FR of the EBV’s *oriP* region and insertion of the *Ava*I 1,437 bp fragment from pBR18 into the *Ava*I site to obtain a plasmid of 6,126 bp; 2) pAC_EBV*oriP*, a derivative of pHEBo, supplied by J. Yates [24] obtained after removal of the *Sal*I-*Ssp*I 842 bp fragment containing no relevant elements; 3) pEco3’Δ; and 4) pCXWB-EBNA1 (Addgene number: #37624) containing the coding region of EBNA1.

### Bacterial strains and cell cultures

The *E*. *coli* strain used was DH5αF′ {F′*/gyrA96*(Nal^*r*^) *recA1 relA1 endA1 thi-1 hsdR17* (*r*_*k*_^−^*m*_*k*_^+^) *glnV44 deoR* Δ(*lacZYA-argF*) *U169*[F80dΔ(*lacZ*)*M15*]}. Competent cells were transformed with monomeric forms of pAC_SV40*ori*, pAC_EBV*oriP* or pEco3’Δ. Transformed DH5αF′ cells were grown in LB medium with 75 μg/ml of ampicillin at 37°C. Isolation of plasmid DNA was performed using a plasmid DNA purification kit (Macherey-Nagel).

Original Human Embryonic Kidney (HEK) 293 cells and variants cells that express either the T-antigen (HEK 293T) or the EBNA-1 protein (HEK 293E), were cultured in Dulbecco’s modified Eagle's medium (DMEM) supplemented with 10% fetal bovine serum (FBS), 100 units/ml of penicillin, 100 μg/ml streptomycin (Gibco) and L-glutamine 2 mM (Gibco).

### HEK 293 cell transfection and HIRT episome extraction

A total of 1× 10^6^ HEK 293, HEK 293E or HEK 293T cells were plated per 10 cm diameter dish 1 day before transfection. Cells were transfected or co-transfected with 6 μg of monomeric forms of pAC_SV40*ori*, pAC_EBV*oriP*, pEco3’Δ and/or pCXWB-EBNA1 plasmids in antibiotic free media using the Lipofectamine 2000 reagent (Life Techologies). Media was changed 24 h after transfection. 40 h after transfection, plates were rinsed with 1× PBS and the cells washed off the plate with ice-cold PBS, washed again with ice-cold PBS and HIRT extracted as described Scully et al. [25].

### Two-dimensional agarose gel electrophoresis and Southern transfer

The first dimension was in a 0.4% agarose (Seakem®LE, Lonza) gel in TBE buffer (89 mM Tris borate, 2 mM EDTA) at 0.9 V/cm at room temperature for 25 h. The second dimension was in a 1% agarose gel in TBE buffer and was run perpendicular to the first dimension. The dissolved agarose was poured around the excised agarose lane from the first dimension and electrophoresis was at 5 V/cm in a 4°C cold chamber for 12 h. When necessary, different concentrations of chloroquine (Sigma) were added to the TBE buffer in both the agarose gel and the running buffer in the first dimension. Southern transfer was performed as described before [26–28].

### Non-radioactive hybridization

DNA probes were labeled with digoxigenin using the DIG-High Prime kit (Roche Applied Science). Membranes (Zeta-Probe GT membranes, Bio-Rad) were pre-hybridized in a 20 ml pre-hybridization solution (2× SSPE, 0.5% Blotto, 1% SDS, 10% dextran sulfate, and 0.5 mg/ml sonicated and denatured salmon sperm DNA) at 65°C for 4–6 h. Labeled DNA was added, and the hybridization lasted for 12–16 h. Hybridized membranes were sequentially washed with 2× SSC and 0.1% SDS at room temperature for 5 min twice and with 0.1× SSC and 0.1% SDS at 68°C for 15 min twice as well. Detection was performed with an antidigoxigenin-alkaline phosphatase-conjugated antibody (Roche Applied Science) and CDP-Star (PerkinElmer Life Sciences) according to the instructions provided by the manufacturers as described before [29]. All experiments were performed twice to confirm the results obtained were reproducible.

### Immunoblot analysis

HEK 293T were lysed with NP-40 buffer (20 mM Tris-HCl pH 7.5, 10% glycerol, 137 mM NaCl, 1% NP-40, 1 mM sodium orthovanadate, 10 mM sodium fluoride, EDTA 2 mM) containing protease inhibitors (Sigma). Proteins were separated by 12% SDS-polyacrylamide gel electrophoresis and transferred to a PVDF membrane (Biorad). The membranes were incubated with mouse monoclonal anti-EBV EBNA-1 antibody (Santa Cruz) and rabbit polyclonal anti-αTubulin (ABclonal), followed by HRP-conjugated anti-mouse (Santa Cruz) or anti-rabbit IgG (DAKO).

## Results

**Fig 2:**
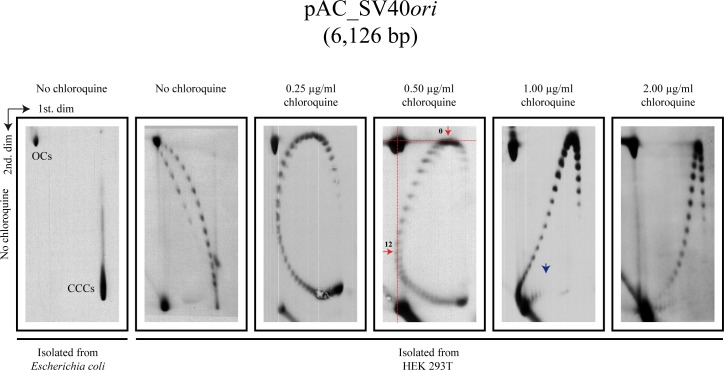
Intact forms of pAC_SV40*ori* isolated from *E*. *coli* and HEK 293T cells analyzed by two-dimensional agarose gel electrophoresis (2D gels) where the first dimension occurred without or in the presence of different concentrations of chloroquine. The electrophoretic mobility of topoisomers with **ΔLk** = 0 during the first and second dimensions are indicated by horizontal and perpendicular broken red lines in the panel run with 0.50 μg/ml chloroquine. The blue arrow points the transition to a non-B conformation in the 2D gel where the first dimension occurred in the presence of 1.00 μg/ml chloroquine.

**Fig 3:**
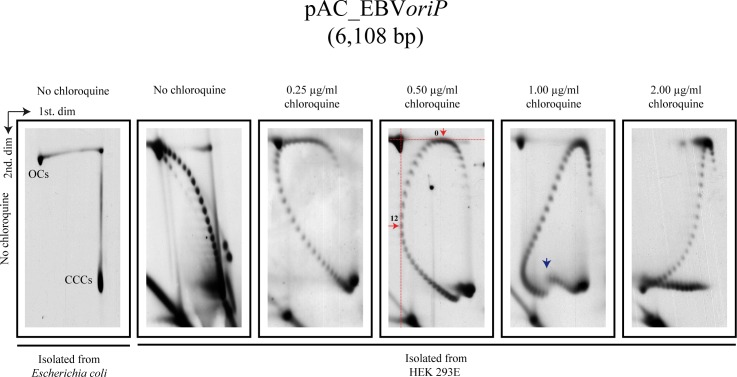
Intact forms of pAC_EBV*oriP* isolated from *E*. *coli* and HEK 293E cells analyzed by two-dimensional agarose gel electrophoresis (2D gels) where the first dimension occurred without or in the presence of different concentrations of chloroquine. The electrophoretic mobility of topoisomers with ΔLk = 0 during the first and second dimensions are indicated by horizontal and perpendicular broken red lines in the panel run with 0.50 μg/ml chloroquine. The blue arrow points the transition to a non-B conformation in the 2D gel where the first dimension occurred in the presence of 1.00 μg/ml chloroquine.

First, we transfected HEK 293T and HEK 293E cells with covalently-closed circles (CCC) of pAC_SV40*ori* or pAC_EBV*oriP*, respectively, isolated from *E*. *coli* cells. Transfected cells were cultured in appropriate media and extrachromosomal DNA was HIRT extracted 40 hours later. These DNAs were first analyzed in 2D gels run in the absence of chloroquine (second to far left panels in Figs 2 and 3). Both (+) and (-) supercoiled forms were clearly detected for pAC_SV40*ori* and pAC_EBV*oriP* isolated from HEK 293 cells. For comparison, the far left panels in both figures corresponded to the same DNA molecules isolated from *E*. *coli* cells. Here only heavily negatively supercoiled (CCCs) and nicked molecules (OCs) were detected.

**Fig 4:**
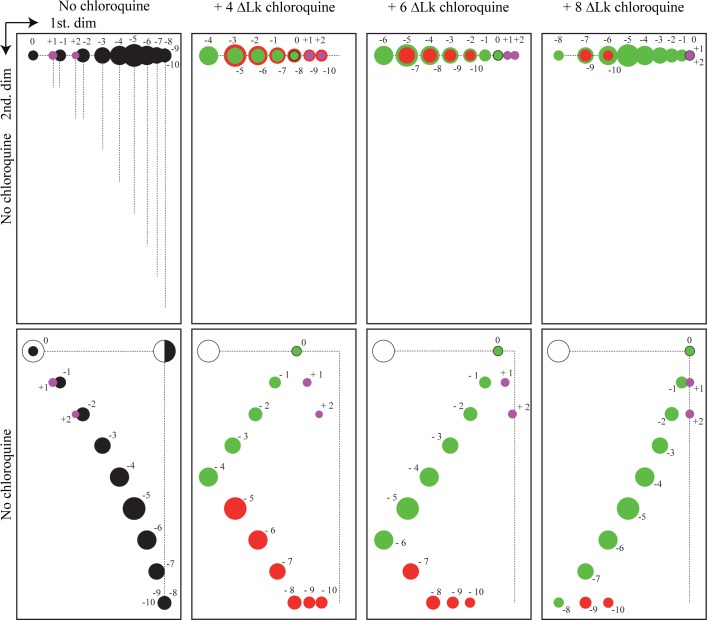
Cartoons describing in a diagrammatic manner the behavior of (-) and (+) supercoiled topoisomers in 2D gels without or where the first dimension occurred in the presence of different concentrations of an intercalator. We consider a theoretical population of 13 topoisomers with a ΔLk ranging from +2 to -10 (indicated as such in all diagrams), with the modal topoisomer being ΔLk = -5. The diameter of the circles indicates their abundance in the population. To simplify the model, we established that in these 2D gels resolution would go from ΔLk = 0 to ΔLk = +/- 8 (in real 2D gels resolution goes from ΔLk = 0 to ΔLk = ~ +/- 20). Diagrams to the far left show the electrophoretic mobility of these topoisomers after the first (top) and second (bottom) dimensions run in the absence of intercalating drugs. Vertical broken lines highlight the mobility of each topoisomer after the first and second dimensions. Second left diagrams show their electrophoretic mobility when the first dimension occurred in the presence of an intercalator that introduced four (+) turns to every topoisomer. Diagrams second to right show their electrophoretic mobility when the first dimension occurred in the presence of an intercalator that introduced six (+) turns to every topoisomer. Finally, far right diagrams show their electrophoretic mobility when the first dimension occurred in the presence of an intercalator that introduced eight (+) turns to every topoisomer. Each filled circle represents individual topoisomers. An empty circle corresponds nicked circles. The half black circle represents a topoisomer that migrated as heavily supercoiled (CCC) during the first dimension, was nicked between the first and second dimensions and migrated as a relaxed molecule (OC) during the second dimension. Color code: black circles represent topoisomers that migrated as (-) supercoiled during the first and second dimensions in the absence of chloroquine. Green circles represent those topoisomers that turned (+) supercoiled in the presence of the intercalator during the first dimension. Red circles represent those topoisomers that remained (-) supercoiled in the presence of the intercalator during the first dimension. Note that their number diminished as the (+) ΔLk introduced by the intercalator increased. Violet circles represent those topoisomers that remained (+) supercoiled during the first and second dimensions. Note that after the second dimension run, highly (-) supercoiled topoisomers appear at the bottom whereas (+) supercoiled topoisomers appear at the top right.

In order to distinguish all the topoisomers in both populations we run 2D gels where the first dimension occurred in the presence of different concentrations of chloroquine. Fig 4 describes in a diagrammatic manner the behavior of (-) and (+) supercoiled topoisomers in 2D gels where only the first dimension occurred in the presence of different concentrations of the intercalator. This experimental protocol allows identification of the number of (+) turns introduced by an intercalator in each case. It corresponds to the number of topoisomers that lay between the topoisomer that migrated with a ΔLk = 0 after the first dimension and the topoisomer that migrated with a ΔLk = 0 after the second dimension. Note also that the most (-) supercoiled topoisomers migrate to the bottom (indicated in red in Fig 4) whereas the (+) supercoiled ones migrate close to the top right corner (indicated in magenta in Fig 4).

**Fig 5:**
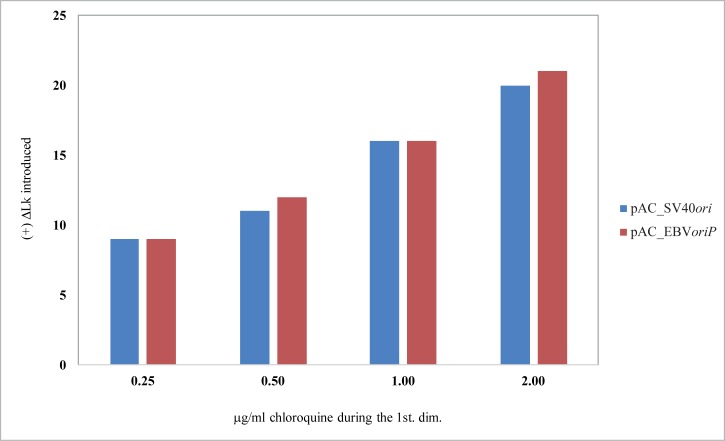
Histogram showing the efficiency of different concentrations of chloroquine to introduce (+) ΔLk in pAC_SV40*ori* (6,126 bp) and pAC_EBV*oriP* (6,108 bp). The number of (+) ΔLk introduced by the different concentrations of chloroquine corresponds to the number of topoisomers that lay between the topoisomer that migrated with a ΔLk = 0 after the first dimension and the topoisomer that migrated with a ΔLk = 0 after the second dimension (see broken red lines in Figs 2 and 3). Note that the net number of (+) supercoils introduced was almost identical in both cases.

We used the results obtained for both minichromosomes to determine the number of (+) ΔLk introduced by the different concentrations of chloroquine used. To identify the topoisomer that run with a ΔLk = 0 after the first and second dimensions we used as a guide the electrophoretic mobility of the nicked (OC) forms (indicated by red broken lines in the panel exposed to 0.50 μg/ml chloroquine during the first dimension in Figs 2 and 3). The results were compared in a histogram shown in Fig 5. Note that 0.25 μg/ml chloroquine introduced ~ 9 (+) turns in both cases, 0.50 μg/ml introduced ~ 11 (+) turns in pAC_SV40*ori* and ~ 12 (+) turns in pAC_EBV*oriP*, 1.00 μg/ml introduced ~ 16 in both minichromosomes and 2.00 μg/ml introduced ~ 20 and ~ 21 (+) turns, respectively. We concluded that chloroquine’s efficiency to introduce (+) turns in both minichromosomes was almost identical.

Comparison of the panel corresponding to 2D gels exposed to 1.00 μg/ml chloroquine during the first dimension revealed another interesting feature. It is well known that in the case of (-) supercoiled DNAs, heavily supercoiled forms adopt a non-B DNA conformation that is usually accompanied by the sudden appearance of single-stranded stretches at a precise ΔLk that reduces electrophoretic mobility [18, 30]. Transitions to a non-B conformation (indicated by a vertical blue arrow in Figs 2 and 3) were clearly observed in the 2D gel panel when 1.00 μg/ml chloroquine was used during the first dimension. These transitions occurred in topoisomers with specific a ΔLk. In the first dimension in the presence of chloroquine, non-B DNA conformations are inhibited (as the overall torsion in the DNA is reduced). Therefore, no discontinuity occurred in the first dimension in the presence of chloroquine. When the second dimension was carried out in the absence of chloroquine, though, the DNA recovered its original (-) torsion and the acquisition of a non-B conformation became apparent as a sharp shift upwards in the vertical dimension (pointed with blue vertical arrows in the corresponding panel in Figs 2 and 3). These observations confirmed that the native supercoiling density of pAC_SV40*ori* and pAC_EBV*oriP* was almost identical.

To further confirm this observation and shed new light on its possible cause we decided to analyze the electrophoretic mobility of topoisomers of another minichromosome: pEco3’Δ in the absence and in the presence of EBNA-1. pEco3’Δ contains the SV40 replication origin and the FR of EBV’s *oriP* (see Fig 1). As the DS element is missing in this minichromosome, *oriP* cannot function as a replication origin even when EBNA-1 is provided *in trans* [23, 31]. In consequence, pEco3’Δ cannot replicate in HEK 293E cells but it replicates efficiently in HEK 293T cells. It was clearly demonstrated by *in vitro* assays, that in this minichromosome initiation of DNA replication takes place bi-directionally at the SV40 origin and the EBV’s FR acts as a replication fork barrier (RFB) and termination site provided EBNA-1 is added *in trans* [23, 31].

**Fig 6:**
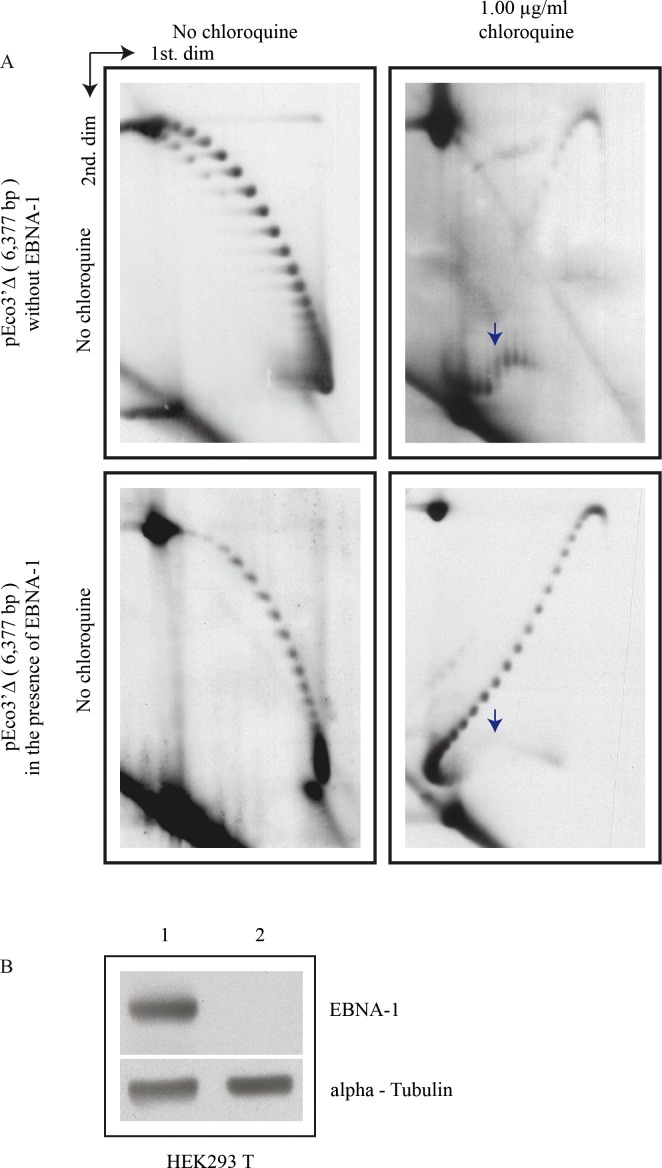
Intact forms of pEco3’Δ isolated from HEK 293T cells in the absence or presence of EBNA-1 analyzed by two-dimensional agarose gel electrophoresis (2D gels) and their corresponding Western blots. **(A)** Note that topoisomers with (-) and (+) supercoiling were clearly distinguished. The panel shown on top corresponds to DNA isolated from cells without EBNA-1. Those shown below were isolated from cells in the presence of EBNA-1. Panels to the left were analyzed in 2D gels run without chloroquine whereas those to the right were analyzed in 2D gels where the first dimension occurred in the presence of 1.00 μg/ml chloroquine. The blue arrows point the transition to a non-B DNA conformation. **(B)** Total cellular proteins from HEK 293T cells co-transfected with pEco3’Δ and pCXWB-EBNA1 (column 1) and from the same cell line transfected with pEco3’Δ alone (column 2) subjected to Western blotting for EBNA-1 and α-Tubulin.

We transfected HEK 293T cells with pEco3’Δ alone or co-transfected them with the same minichromosome and pCXWB-EBNA1, another minichromosome that expresses EBNA-1. The transfected cells were cultured as in the previous cases and extrachromosomal DNA was HIRT extracted 40 hours later. DNA samples were analyzed in 2D gels run without chloroquine as well as with 1.00 μg/ml chloroquine only during the first dimension. Transitions to a non-B conformation (indicated by vertical blue arrows in both 2D gel panels where 1.00 μg/ml chloroquine was used during the first dimension) were clearly observed regardless of EBNA-1 (see far right panel in Fig 6A).

To confirm the presence of EBNA-1 in those cells co-transfected with pEco3’Δ and pCXWB-EBNA1, proteins were isolated from cells of the same culture used for DNA analysis. The immunoblot at the bottom left corner in Fig 6B showed that EBNA-1 was present in the co-transfected HEK 293T cells (analyzed in column 1) whereas no traces of this antigen occurred in those cells transfected with pEco3’Δ alone (analyzed in column 2).

**Fig 7:**
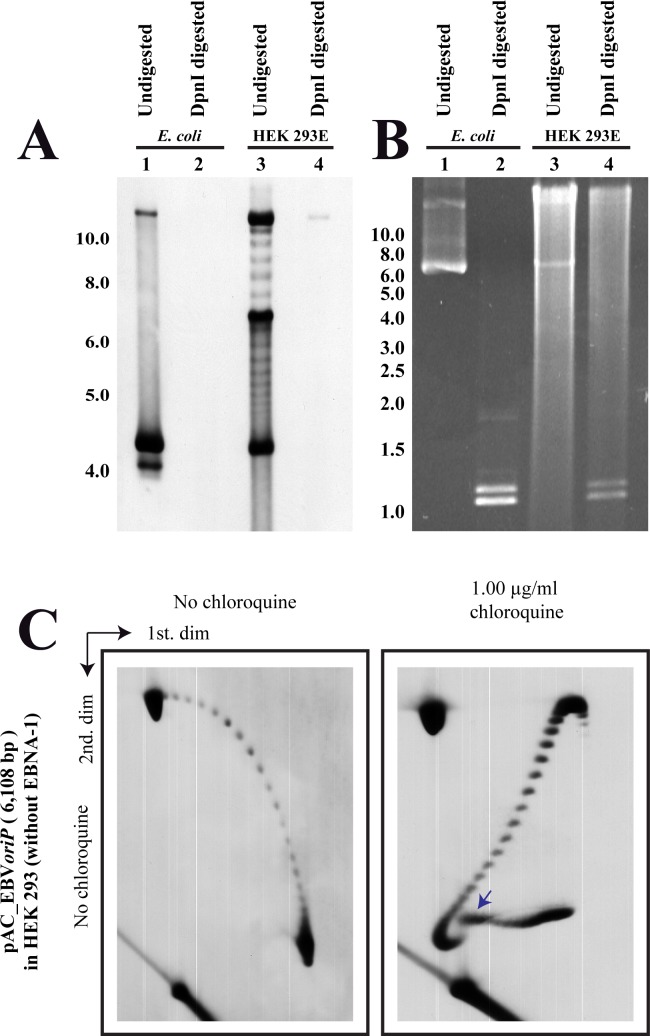
Most of pAC_EBV*oriP* DNA transfected into HEK 293E cells remained unreplicated up to 40 hours after transfection. pAC_EBV*oriP* DNA isolated from *E*. *coli* and from HEK 293E cells 40 hours after transfection was analyzed intact and after digestion with DpnI. Selected non-radioactive hybridization (A) and ethidium bromide staining (B) showed that in both cases DpnI digested almost all the DNA. In addition, DNA of the minichromosomes isolated from HEK 293 cells (that do not express EBNA-1) were analyzed in 2D gels run without chloroquine (to the left) and in 2D gels where the first dimension occurred in the presence of 1.00 μg/ml chloroquine (C). As these minichromosomes were isolated from HEK 293 cells that do not express EBNA-1, they cannot possibly support *oriP* initiation of replication. The blue arrows point the transition to a non-B DNA conformation.

Finally, we checked if the minichromosomes replicated in the transfected cells. To this end we used pAC_EBV*oriP* DNA isolated from *E*. *coli* cells and from HEK 293E cells 40 hours after transfection. These DNAs were analyzed undigested and after digestion with DpnI. The results obtained are shown in Fig 7A and 7B. In both cases, DpnI digested almost all the DNA indicating that most of the DNA transfected into HEK 293E cells did not replicate in the human cells even though the initiator protein, EBNA-1, was present. To confirm that the supercoiling patterns observed for pAC_EBV*oriP* were solely due to the assembly of chromatin and not to the viral origin binding protein, we used the same minichromosome to transfect the original HEK 293 cells that do not express EBNA-1. Transfected cells were cultured in appropriate media and extrachromosomal DNA was HIRT extracted 40 hours later. This DNA was analyzed in 2D gels run without chloroquine and with 1.0 μg/ml chloroquine only during the 2D gel’s first dimension. The results obtained were very similar to those shown in Fig 3.

In summary, altogether these observations clearly indicated that the presence of EBNA-1 in the transfected cells and its putative binding to the EBV’s *oriP* has no apparent topological consequence.

## Discussion

Initiation of DNA replication is a fascinating subject still not fully characterized. There are countless models and numerous reviews in the literature and many laboratories around the world keep investigating this matter from different perspectives [32–34]. Nonetheless, the model proposed for the *E*. *coli* oriC replication origin is still one of the most popular [35]. In this model, binding of the dnaA protein to the four 9-mers enhances the (-) supercoiling of the region and promotes melting of the three 13-mers. The dnaB-dnaC complex can then get in to extend the duplex opening and generate a pre-priming complex. This model was adapted to explain initiation of DNA replication at the *Sacharomyces cerevisiae* ARS1 origin [36] and it is still used as a guide to explain the first steps in the initiation of DNA replication in different systems. The basic idea is that binding of a protein to specific DNA sequences increases the torsional tension of the region and causes A+T rich sequences nearby to melt. This allows the replication machinery to bind single-stranded chains to initiate the replication process.

Contrary to the situation in prokaryotes, it is well known that in eukaryotes, about 146 bp of DNA are wrap in a 1.6 left-handed superhelical turn around protein octamers consisting of histones named nucleosomes. In covalently-closed circular DNA molecules, when these nucleosomes are removed the DNA has a linking difference (ΔLk) corresponding to approximately -1 per nucleosome removed [18].

The Epstein-Barr virus (EBV) is a paradigm for human tumor viruses. It causes both lymphomas and carcinomas; yet these tumors arise infrequently given that most people in the world are infected with the virus [3]. Binding of EBNA-1 to its cognate sites in *oriP* induces DNA to bend significantly [37] and this bending forces DNA to adopt a distorted conformation throughout most of the cell cycle [38]. These observations imply that EBNA-1 binding to its cognate DNA sites could have significant topological consequences. Indeed, it was suggested that the abundance of single-stranded structures detected within *oriP* in the presence but not in the absence of EBNA-1 could be due to transiently unwound regions that form because of an excess of negative supercoiling [9]. EBNA-1, though, was shown to assemble on chromatin containing *oriP* [39]. In other words, the EBNA-1 protein can efficiently access its DNA cognate sites within a nucleosome and does not cause removal or the precise positioning of nucleosomes within or adjacent to the FR and DS elements of *oriP*. These latter observations challenge those aforementioned, suggesting that EBNA-1 binding to DNA could have no significant topological consequences. This is precisely the case for the large T-antigen bound to the SV40 replication origin. This binding is cell cycle specific and is not expected to have topological consequences throughout most of the cell cycle [40]. However, until now no systematic comparison has been reported on the topology of minichromosomes carrying the SV40 origin and the *oriP* replication origin of EBV.

The observations that the FR of *oriP* acts as a RFB and termination site both in human B-lymphocytes *in vivo* [11] as well as *in vitro* in pEco3’Δ and derived plasmids where initiation of DNA replication occurred at varying distances of the FR at the SV40 origin [23, 31] suggest that binding of EBNA-1 to the FR creates a complex structure that cannot be solved by DNA helicases. On the contrary, binding of EBNA-1 to the DS element appears to represent no obstacle for the replication fork to go beyond. These findings together with electron micrographs of *oriP* DNA in the presence of large amounts of EBNA-1 [41, 42] imply the possible formation of a DNA loop between the FR and the DS element driven by EBNA-1 where initiation of DNA replication would take place. The observation that the distance between the DS element and the FR must be maintained within certain limits for *oriP* to function as an origin [43] supports the latter model.

Although minichromosomes containing *oriP* support replication in selected human cell clones when the viral protein EBNA-1 is provided, they are lost from cells during 2 weeks post-transfection [44]. Therefore, it could be the molecules analyzed in Fig 3 might correspond to minichromosomes not assembled with EBNA1. However, replication intermediates of these minichromosomes were unambiguously identified 40 hours after transfection [25]. This observation indicates the at least some minichromosomes assemble with EBNA1 as they would not replicate otherwise. We confirmed that most of the transfected DNA remained unreplicated 40 hours after transfection, as DpnI was able to digest almost all of it (Fig 7A and 7B). Moreover, the supercoiling patterns of pAC_EBV*oriP* without and in the presence of 1μg/ml chloroquine were very similar regardless of the presence of EBNA-1 (Fig 7C).

**Fig 8:**
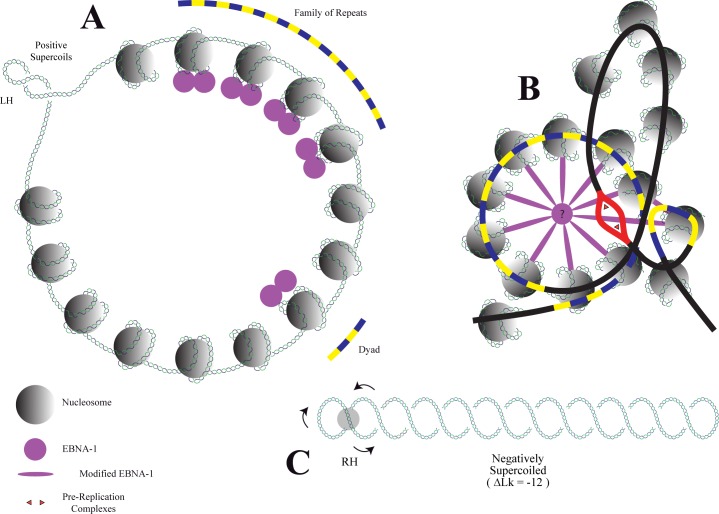
Schematic model illustrating the interaction of EBNA-1 with the DS and the FR of *oriP* in two steps. **A:** In the first step EBNA-1 binds to its cognate DNA sites wrapped around nucleosomes throughout most of the cell cycle. This interaction does not cause removal or the precise positioning of nucleosomes within or adjacent to the FR and DS elements of *oriP*. **B:** In the second step, which is cell cycle specific, an unknown factor (?) causes structural conformation changes that leads to the formation of a loop between the DS and the FR. This conformational change allows the melting of A+T rich regions and initiation of DNA replication close to the DS element. **C:** Once proteins are removed the minichromosome shows a linking difference (ΔLk) corresponding to approximately -1 per nucleosome removed. The gray circle highlights a RH crossing. In the diagram the original molecule shown in (A) would end-up negatively supercoiled with a ΔLk = -12. The relative position of the DS and the FR are shown in yellow and blue and the putative replication initiation site is marked in red. Double-stranded DNA is represented in green and blue.

In summary, here we showed that in HEK 293 cells, minichromosomes of almost the same mass carrying either the SV40 or the EBV latent origin of replication showed similar topological features. We demonstrated also that in pEco3’Δ, a hybrid minichromosome that initiates replication at the SV40 origin, binding of EBNA-1 to the EBV’s FR induces no significant change in the level of supercoiling. These observations challenge the idea that binding of EBNA-1 to *oriP* induces (-) supercoiling and favor a model (Fig 8) suggesting that EBNA-1 binds to *oriP* in a two-step process where only the second step initiates structural changes in a cell cycle specific manner [45].
